# High-Frequency Hearing Loss and Decrease in High-Frequency Hearing Thresholds after Positive Airway Pressure Treatment in Patients with Severe OSA

**DOI:** 10.1055/s-0046-1819642

**Published:** 2026-04-30

**Authors:** Hande Arslan, Zeynep Özer, Nevra Güllü Arslan, Gökhan Akgül, Doğukan Özdemir, Dursun Mehmet Mehel

**Affiliations:** 1 Department of Otorhinolaryngology, Samsun City Hospital, Samsun, Turkey; 2 Department of Audiology, Samsun City Hospital, Samsun, Turkey; 3 Department of Pulmonology, Samsun City Hospital, Samsun, Turkey

**Keywords:** audiometry, high-frequency audiometry, obstructive sleep apnea syndrome, positive airway pressure

## Abstract

**Introduction:**

Obstructive sleep apnea (OSA) is a breathing disorder that causes hypoxia which, in turn, causes cochlear dysfunction.

**Objectives:**

To determine whether Positive Airway Pressure (PAP) treatment has a positive or negative effect on hearing during 6-month follow-up of severe OSA.

**Methods:**

There were 59 patients with severe OSA in the study group and 31 volunteers in the control group. Audiologic tests were performed on both groups before the start of PAP treatment, at the 3 ^rd^ and 6 ^th^ month of the PAP treatment.

**Results:**

The mean ages of the study and control groups were 46.3 ± 7.1 and 43.8 ± 7.7 years, respectively ( *p* = 0.39). The test means for Sleep Efficiency (89.3 ± 4.2), AHI (45.3 ± 16.8), ODI (39.5 ± 20.8), MOS (89.9 ± 3.7), AD (7.6 ± 3.4), LOS (78 ± 8.5), and TDOS (40.7 ± 26) were registered for the study group. High-frequency PTA (HF-PTA) scores were higher in the study group than in the control ( *p* < 0.01); they also decreased in the 3 ^rd^ month of treatment ( *p* < 0.01) and persisted in the 6 ^th^ month. There was a correlation between HF-PTA and MOS, AD, and LOS ( *p* < 0.01; 0.05; 0.045, respectively). Also, 8 kHz hearing levels were highly correlated with HF-PTA in the study and control groups ( *p* < 0.01 and ρ = 0.74; *p* = 0.01 and ρ = 0.58, respectively).

**Conclusion:**

High-frequency hearing threshold levels may show improvement with the use of PAP therapy. Also, the 8 kHz hearing levels may be used to predict high-frequency hearing loss and early cochlear damage.

## Introduction

Obstructive sleep apnea (OSA) is a breathing disorder characterized by the narrowing of the upper airway that impairs normal ventilation during sleep and causes hypoxia. 1 Its prevalence has been estimated to be between 5 and 14% in the population. 2

Continuous Positive Airway Pressure (CPAP) is the first-line standard treatment for OSA, but the long-term acceptance or adherence is reported by the literature to be from 50 to 70%. 3 4 Along with side effects, such as nasal discharge, nasal congestion, dry nose, epistaxis, and dry mouth/throat, 5 some patients also suffer from the noise of the CPAP machine. 6 Although few studies in the literature identify side effects, 5 no study has been found investigating the effect of the machine's noise on hearing. Chronic noise exposure is typically limited to hearing loss in the 3 to 6 kHz region of the pure-tone audiogram. 7 To date, studies have shown that the effect of noise-induced hearing loss (NIHL) first became apparent for high-frequency hearing levels (HL) than conventional audiometers. 8

Hypoxia is another factor that affects cochlear function. 9 Previous studies showed impaired hearing functions in patients with OSA due to hypoxia. 10 11 However, none of the studies in the literature mention the cochlear function changes in point of both low and high frequency hearing threshold levels of severe OSA patients after effective Positive Airway Pressure (PAP) therapy. The current research is the first one in the literature evaluating detailed hearing functions in both low and high-frequency hearing threshold levels within the 6-month follow-up period in patients with severe OSA.

The current research aimed to determine whether PAP treatment would benefit cochlear functions by reducing hypoxia or causing harm to cochlear functions due to noise exposure during the 6-month follow-up period in patients using effective PAP therapy for severe OSA.

## Methods

The present is a prospective cohort study, approved by the Institutional Review Board of the Samsun University Medical School, under the decision number 2023 12/23, from June 2023. All patients signed informed consent forms before inclusion. This research involves human participants and complies with the 1964 Helsinki Declaration and its later amendments.

### Patient Selection

A total of 44 patients who were diagnosed with severe OSA and started CPAP therapy were recruited for the study. Those with any comorbid diseases, obesity, or a history of neurotologic diseases (sudden hearing loss, tinnitus, vertigo) were excluded from the study. Patients using CPAP therapy at least 4 hours a day and 5 days a week were accepted as receiving effective treatment. 12 The time of using the CPAP device was confirmed by examining the records in all cases.

The control group was composed of 31 healthy individuals who applied to the ENT department without any symptoms of OSA, such as snoring, day-time tiredness, witnessed to stop breathing, having no comorbid (hypertension, obesity, diabetes mellitus, coronary artery disease [CAD]) or neurotologic diseases.

### Polysomnography

Polysomnography (PSG) was performed with the Embla N7000 series (Natus Medical Inc.). At least 5 hours of records are considered valid. All exam records were interpreted by the same trained specialist. Standard overnight PSG included continuous monitoring with central electroencephalograms, electrooculograms, submental and anterior tibial electromyograms, and electrocardiograms with conventional leads.

Airflow was monitored by oral and nasal cannula, using pressure transducers and thermistors. Respiratory inductance PSG was performed to measure the degree of respiratory effort, with the transducers placed around the chest and abdomen. The oxyhemoglobin saturation was recorded continuously by pulse oximetry. 13 14 Variables considered in the PSG were apnea-hypopnea index (AHI), oxygen desaturation index (ODI, >4%), mean oxygen saturation (MOS), average desaturation (AD), lowest oxygen saturation (LOS), total duration of oxygen saturation (SpO _2_ ) with less than 90% (TDOS), as recommended by the American Academy of Sleep Medicine (AASM). 1

The OSA was defined in events per hour, as mild (AHI 5–15), moderate (AHI 15–30), and severe (AHI ≥ 30). Patients with PSG results of AHI ≥30 were included in the study.

### Audiological Examination

After middle ear pathologies were excluded by otologic examination and tympanometry test (GSI Tympstar Pro, Grason-Satnadler), transient-evoked otoacoustic emission (TEOAE; Madsen Capella 2, Otometrics) responses at 1.0, 2.0, 3.0, and 4.0 kHz with click stimuli at 80 dB nHL transmitted via inserted earphone and pure tone audiometry test (AC 40 Clinical Audiometer; Interacoustics) between 500 and 18,000 Hz frequencies were performed in a soundproof cabin.

The speech discrimination scores (SDS) were detected at an easily detectable HL by 50 selected monosyllabic words and calculated by the percentage of words correctly identified. Pre- and posttreatment HLs were analyzed. The pure tone average (PTA) was calculated by arithmetic mean for frequencies of 500 to 8000 Hz, and a high-frequency PTA (HF-PTA) was also calculated for frequencies of 10 to 18 kHz. Also, the arithmetic mean for frequencies of 500 to 4000 Hz was called PTA without 8 kHz, and 8 kHz HL were examined separately. Audiologic tests were performed on patients with severe OSA before starting to use the CPAP treatment, at 3 months of treatment, and at 6 months of treatment.

### Statistical Analysis

The analysis of the results was performed using the IBM SPSS Statistics for Windows (IBM Corp.), version 21.0. Data analysis for normal distribution used the Kolmogorov-Smirnov test. The categorical variables were shown as number and percentages, while the normally distributed continuous variables were shown as mean ± standard deviation (SD). Median values (minimum-maximum) were used for the continuous variables that were not normally distributed. Student's *t* -test was used for the analysis of normally distributed continuous variables and the χ2 test for the categorical variables. The Mann-Whitney U test was used for the variables which were not normally distributed.

When > 2 groups were compared, differences among the groups were tested using analysis of variance (ANOVA) for normally distributed variables, the Kruskal-Wallis H test for non-normally distributed ones. Furthermore, Fisher's exact or Pearson's chi-squared tests were used for categorical variables. A *p* -value of 0.05 was considered statistically significant.

Subsequent pair-wise comparisons were performed by two-way repeated measures ANOVA. Pearson's and Spearman's correlation coefficients were used for correlation analysis where appropriate. The 2-way ANOVA variance analysis was performed by using GPower 3.1 (G*Power), version 3.1.9.2, for power analysis. Based on Cohen's effect size coefficients, with an effect size f = 0.69 and a Type I error level of α = 0.05, the actual power was 1.

## Results

A total of 90 individuals were included in the study. There were 59 patients in the study group (male: *n* = 44, 74.6%; female: *n* = 15, 25.4%) and 31 volunteers in the control group (male: *n* = 21, 67.7%; female: *n* = 10, 32.3%). The mean ages of the study and control groups were 46.3 ± 7.1 years and 43.8 ± 7.7 years, respectively. There was no statistically significant difference between the groups in terms of sex and age ( *p* = 0.33 and 0.14, respectively).

The resulting means and SDs of the Sleep Efficiency (89.6 ± 4.1), AHI (37.9 ± 15.7), ODI (43.7 ± 15.7), MOS (89.6 ± 3.7), AD (7.5 ± 3.6), LOS (78.3 ± 8.4), and TDOS (40.5 ± 22) were registered for the study group.

The audiological test results of the study and control groups are presented in Table 1 . Both PTA and HF-PTA scores were higher in the study group than in the control group ( *p* = 0.04, < 0.01; respectively). There were no statistically significant differences in TEOAE levels and speech discrimination scores between the study and control groups ( *p* = 0.45; and 0.37, respectively). The 8 kHz HLs were highly correlated with HF-PTA in both the study ( *p* < 0.01; ρ = 0.68) and control groups ( *p* = 0.01; ρ = 0.59). There were no significant differences in PTA without 8 kHz between the study (14.1 ± 4.2) and control (12.9 ± 2.5) groups ( *p* = 0.11).

**Table 1:** Audiologic test results of control and study groups

	Study (n: 59)	Control (n:31)	*p* -value
**PTA**	16.1 ± 4.6	13.9 ± 3.7	0.04
**HF-PTA**	40.9 ± 21.2	16.5 ± 14	< 0.01
**TEOAE**	9.3 ± 3.8	9.9 ± 2.3	0.45

**Abbreviations:** HF-PTA, high-frequency pure tone average; PTA, pure tone average; TEOAE, transient-evoked otoacoustic emissions.

The audiology test results of the study group before the start of CPAP treatment, at 3 months, and at 6 months were summarized in Table 2 . According to the results, HF-PTA scores decreased in the 3 ^rd^ month of treatment, and the means of PTA and HF-PTA scores remained unchanged in the 6 ^th^ month.

**Table 2:** Effect of PAP treatment on audiologic test results in the study group

	PTA	HF-PTA	PTA-3	HF-PTA-3	PTA-6	HF-PTA-6	*p* -value
**Study group**	16.1 ± 4.6	40.9 ± 21.2	17 ± 5.7	36.7 ± 20.3	17.1 ± 5.8	37.7 ± 20.9	0.002* 0.004 ^a^ <0.001 ^b^ 0.93 ^c^ 0.79 ^d^

**Abbreviations:** HF-PTA, high-frequency pure tone average; PAP, positive airway pressure; PTA, pure tone average; PTA-3, pure tone average in the 3 ^rd^ month of PAP treatment; PTA-6, pure tone average in the 6 ^th^ month of PAP treatment; TEOAE, Transient-evoked otoacoustic emissions. **Notes** : *PTA versus PTA-3; ^a^ PTA versus PTA-6; ^b^ HF-PTA versus HF-PTA-3; ^c^ PTA-3 versus PTA-6; ^d^ HF-PTA-3 versus HF-PTA-6.

The correlation between PSG parameters and the audiology test results (PTA, HF-PTA, 8 kHz HL, and PTA without 8 kHz) are shown in Table 3 .

**Table 3:** The correlations between PSG parameters and audiologic test results in the study group

	AHI	ODI	MOS	AD	LOS	TDOS	
**PTA**	0.04	0.06	0.02	0.02	0.03	0.1	***p-*** **value**
	0.3	0.25	−0.31	0.31	−0.28	0.21	**ρ value**
**HF-PTA**	0.01	0.02	< 0.001	0.03	0.04	0.01	***p-*** **value**
	0.3	0.14	−0.45	0.15	−0.41	0.38	**ρ value**
**8 kHz HL**	0.27	0.14	0.015	0.04	0.09	0.09	***p-*** **value**
	0.17	0.23	−0.36	0.32	−0.25	0.06	**ρ value**
**PTA without 8 kHz**	0.25	0.29	0.14	0.27	0.39	0.27	***p-*** **value**
	0.18	0.16	−0.23	0.17	−0.13	0.17	**ρ value**

**Abbreviations:** 8 kHz HL, 8 kHz hearing Level; AD, average desaturation; AHI, apnea-hypopnea index; HF-PTA, high-frequency pure tone average; LOS, lowest oxygen saturation; MOS, mean oxygen saturation; ODI, oxygen desaturation index >4%; PSG, polysomnography; PTA, pure tone average; TDOS, total duration of oxygen saturation with less than 90%. **Note** : A *p* < 0.05 is statistically significant, *p* -value is the rho value showing the correlation coefficient.

## Discussion

In this study, we have demonstrated that cochlear functions got worse in patients with severe OSA. For the first time in the literature, high-frequency hearing thresholds were shown to be elevated in patients with severe OSA, and the effective CPAP treatment caused a decrease in the high-frequency hearing threshold levels at the 3 ^rd^ month of treatment, also persisting at 6-months. In addition to these findings, it was shown that the hearing thresholds of 8 kHz were strongly correlated with the HF-PTA scores in both healthy individuals and OSA patients.

The noise of the CPAP machine is a challenging matter for patients during therapy. A previous study demonstrated that, on average, a 30 dB broadband noise is generated from CPAP machine, and the most critical location of noise generation is the fan region and fan inlet. 15 Chronic noise exposure is typically limited to hearing loss in the 3 to 6 kHz thresholds of the pure-tone audiogram. 7 According to current research results, there was no worsening in the patients using effective CPAP therapy in PTA scores between 500 and 8,000 Hz. When we evaluated high-frequency hearing thresholds to avoid missing early cochlear damage from chronic noise exposure, no deterioration was detected. On the contrary, the improvement was seen in the high-frequency hearing threshold levels in the patients with severe OSA.

Hypoxia is a well-described factor that adversely affects cochlear functions in both animal and human studies. 11 16 However, in the speech frequencies, the adverse effect of hypoxia could not be detected in both pure tone audiograms and TEOAEs. 17 18 In line with the literature, in the current research, we didn't find any differences in the hearing thresholds and TOAES in the speech frequencies between severe OSA patients and healthy individuals. A recently published review also mentioned the lack of studies on hearing threshold levels of extended high frequencies in patients with severe OSA. 19 In the current research, beginning from 8 kHz, the statistically significant difference was shown in the high-frequency thresholds (10–18 kHz) between severe OSA patients and healthy participants, so the hypothesis of high-frequency hearing loss in the patients with severe OSA was supported. To confirm these findings, further studies should be planned to prove the spiral ganglion neurons are more sensitive to hypoxia than the middle and apical turns at the basal scala tympani, which is the high-frequency hearing zone. 19

In addition to indicating cochlear dysfunction, high-frequency hearing loss has been clinically shown to cause a decrease in speech discrimination in noisy environments, sound localization and cognitive impairment. 20 21 However, most of these studies have investigated only 6,000 and 8,000 Hz for the high frequencies. Further studies are needed on the clinical effects of extended high-frequency hearing loss (speech understanding in noise, cognitive function, sound localization, tinnitus) at frequencies above 8,000 Hz.

A systematic review found that hearing threshold levels were significantly poorer among participants with OSA (especially in severe cases) than non-OSA controls. Besides CPAP treatment for OSA did not show improvement in audiometry-measured hearing loss. 22 The first study cited in the systematic review showed improvement in PTA thresholds at 500 to 8,000 Hz frequencies after 3 months of CPAP therapy, and the improvement persisted throughout the 6 months and the 1 ^st^ year. 23 The study group consisted of 28 patients with sensorineural hearing loss (SNHL) and the authors evaluated those who were using CPAP therapy for sleep-disordered breathing. 23

The second study cited in the systematic review consisted of 20 patients with Ménière's disease, and showed that the comorbidity of OSA in these patients exacerbates the underlying condition, and that treating it can reduce disease morbidity. 24 Both two studies evaluated the CPAP therapy in hearing threshold levels indirectly in patients with neurotologic disorders. Also, the HF-PTA (10–18 kHz) test was not performed in either the first or second studies.

Another study showed that CPAP devices used in the treatment of OSA didn't cause positive or negative effects on hearing, when compared with patients with no CPAP use. 25 The study group consisted of 22 patients with moderate-severe OSA but the frequencies of PTA test weren't cleared in the methods section. 25 To our knowledge, the current research is the first one evaluating the effect of CPAP therapy in both low and high-frequency hearing threshold levels in severe OSA patients without any neurotologic diseases.

Studies over the past few years have attempted to determine normative values for hearing thresholds obtained with extended high-frequency audiometry. 26 27 Therefore, it is currently not possible to determine the degree of hearing loss occurring at these frequencies. Because the hearing thresholds in our study were assessed for the same individuals, we consider the decrease here to be significant. However, comprehensive studies on extended high-frequency audiometry are needed.

In patients with other potential confounding factors that may affect hearing (e.g., occupational/hobby noise exposure, genetic predisposition, ototoxicity) and comorbid diseases, a hearing test including high frequency can be performed in addition to conventional audiometry testing, to determine in advance whether cochlear functions are affected. 28

The test for high-frequency hearing threshold levels is a time-consuming task for audiologists in a daily routine. 24 For this reason, we evaluated 8 kHz hearing thresholds separately and a strong correlation was detected between them and PTA scores of high-frequency hearing threshold levels. As such, 8 kHz may be used to predict high-frequency hearing loss.

## Limitations of the Study

The PTA test is a subjective test for evaluating hearing threshold levels. For levels in speech frequencies, the TEOAE test may be enough to confirm PTA test, but the number of objective tests is limited, especially to evaluate high-frequency hearing threshold levels. Due to the lack of any objective test to evaluate high-frequency hearing threshold levels in our clinic, we could not prove these results objectively.

## Conclusion

High-frequency hearing loss is another comorbidity in patients with severe OSA, most often caused by hypoxia, which may show improvement with the use of PAP therapy. Furthermore, clinicians may easily use 8 kHz hearing threshold levels to predict high-frequency hearing loss.
